# The FUS/circEZH2/KLF5/ feedback loop contributes to CXCR4-induced liver metastasis of breast cancer by enhancing epithelial-mesenchymal transition

**DOI:** 10.1186/s12943-022-01653-2

**Published:** 2022-10-12

**Authors:** Peng Liu, Zehao Wang, Xueqi Ou, Peng Wu, Yue Zhang, Song Wu, Xiangsheng Xiao, Yuehua Li, Feng Ye, Hailin Tang

**Affiliations:** 1 Department of Breast Oncology, State Key Laboratory of Oncology in South China, Sun Yat-Sen University Cancer Center, Guangzhou, China; 2 Department of Medical Oncology, the First Affiliated Hospital, Hengyang Medical School, University of South China, Hengyang, China

**Keywords:** circRNAs, Breast cancer, Metastasis, Feedback loop, EMT

## Abstract

**Background:**

Metastasis of breast cancer have caused the majority of cancer-related death worldwide. The circRNAs are associated with tumorigenesis and metastasis in breast cancer according to recent research. However, the biological mechanism of circRNAs in liver metastatic breast cancer remains ambiguous yet.

**Methods:**

Microarray analysis of three pairs of primary BC tissues and matched hepatic metastatic specimens identified circEZH2. We used RT-qPCR and FISH assays to confirm circEZH2 existence, characteristics, and expression. Both in vivo and in vitro, circEZH2 played an oncogenic role which promoted metastasis as well. A range of bioinformatic analysis, Western blot, RNA pull-down, RIP, ChIP, and animal experiments were used to define the feedback loop involving FUS, circEZH2, miR-217-5p, KLF5, FUS, CXCR4 as well as epithelial and mesenchymal transition.

**Results:**

In our research, circEZH2 was proved to be upregulated in liver metastases in BC and predicted the worse prognosis in breast cancer patients. Overexpression of circEZH2 notably accentuated the vitality and invasion of BC cells, whereas knockdown of circEZH2 elicited the literally opposite effects. Besides, overexpressed circEZH2 promoted tumorigenesis and liver metastasis in vivo. Moreover, circEZH2 could adsorb miR-217-5p to upregulate KLF5 thus leading to activate FUS transcription which would facilitate the back-splicing program of circEZH2. Meanwhile, KLF5 could upregulated CXCR4 transcriptionally to accelerate epithelial and mesenchymal transition of breast cancer.

**Conclusions:**

Consequently, a novel feedback loop FUS/circEZH2/KLF5/CXCR4 was established while circEZH2 could be novel biomarker and potential target for BC patients’ therapy.

**Supplementary Information:**

The online version contains supplementary material available at 10.1186/s12943-022-01653-2.

## Background

Breast cancer (BC) has been the most prevalent cancer in the population of women which has been the capital reason for cancer-relative female mortality globally. According to the recent research, the overall survival rate for primary BC in 5 years has been nearly 90%. Nevertheless, 33% of BC patients appear non-nodal distant metastases that shorten overall survival rate of 5 years down to 23% [1]. Clinical research revealed that BC metastasized mostly to the lung, bone, and liver via circulatory system, while liver ranks the third among these BC-metastatic organs. On average, half of BC patients with distant metastases develop liver metastases, and about 5–12% of BC patients manifest liver metastases as their primary organ with recurrence [2]. Untreated BC-derived liver metastases (BCLM) have a survival rate of 4 to 8 months approximately [3]. In contrast, recommended therapy for BCLM rely primarily on chemotherapy and/or systemic hormones, which may prolong the survival period of BCLM patients to 18–24 months only [4–6]. Metastatic disseminating tumor cells acquire organ-specific characteristics for adaptation to the targeted-organ microenvironment. Recent research found that low E-cadherin, high Claudin-2 and high fibronectin aided in liver metastatic formation [7, 8]. However, little is discovered around the biological mechanism of circRNAs that wound accentuate the ability of invasion of BC cells in progression.

Only 2% of the cellular transcriptome is transcribed into mRNAs, while the majority is noncoding RNAs [9]. CircRNAs have been validated to be widespread in cytoplasm and nucleus of cells and play an essential role in abundant biological regulations. Compared to linear mRNAs, there are no traditional structures of RNA, such as the 3′-poly-A tail, which endows circRNAs more steady and evolutional conservative [10, 11]. As an intense research spotlight, circRNAs have absorbed attention of many researchers due to their capability to adsorb microRNAs (miRNAs), bind and interact with proteins, or even translate novel proteins [12–14]. According to recent reports, many human cancers have been validated to be associated with aberrant expression in circRNAs [15–18]. All the above findings indicated that circRNAs were essential in progression of cancers and are ideal biomarkers of predicting prognosis and new therapeutic targets. Nevertheless, biological regulatory functions of circRNAs in the accentuation of BCLM has not been elucidated yet.

In our article, a new circRNA hsa_circ_0008324 (circEZH2) was verified via microarray which was upregulated significantly in BCLM and predicted poor survival of BC patients. CircEZH2 enhanced tumorigenesis and metastasis in vitro and vivo by upregulating KLF5 protein expression through sponging with miR-217-5p, while the transcriptional activation of FUS by KLF5 promoted the process of back-splicing of circEZH2. Eventually, KLF5 activated CXCR4 transcription to induce EMT in BC.

## Results

### CircEZH2 associates with BC liver metastasis

**Fig. 1:**
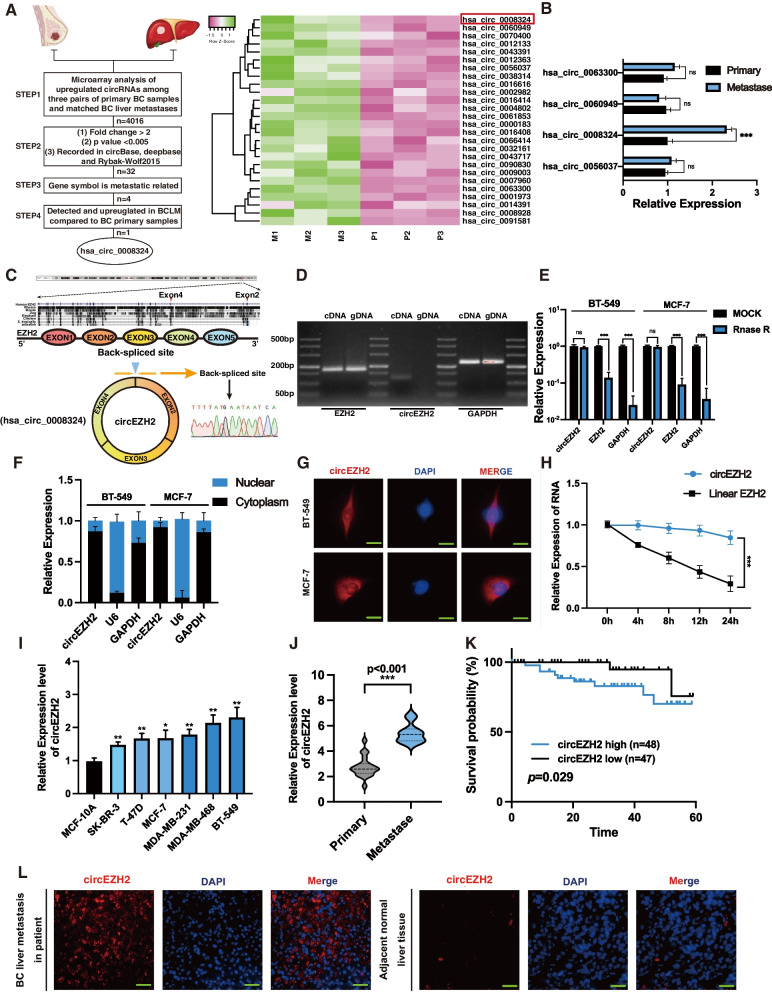
circEZH2 is identified and characterized in BC. **a** Left, flowchart showing the screening criteria of upregulated circRNAs enriched in BCLM; right, heatmap revealing that 32 circRNAs was selected according to the step2 criteria and hsa_circ_0008324 was marked by red box. **b** RT-qPCR was used to testify the expression of four circRNA candidates among BC liver metastases and BC primary tumors. **c** Top, three exons of circEZH2 were highly conservative in different species according to NCBI; bottom, the consitutions of circEZH2 and Sanger-sequencing analysis identified the back-spliced site of circEZH2. **d** Divergent and convergent primers were used for amplification of circEZH2 in cDNA and gDNA while linear EZH2 and GAPDH were used as controls through PCR. **e** Following digestion with Rnase R, RT-qPCR was used to measure the change in circEZH2 and EZH2. **f** Nuclear-cytoplasmic fraction assays were performed to determine the subcellular expression of circEZH2. **g** FISH assays were performed to determine that circEZH2 was localized in the cytoplasm (Scale bar 20 μm). **h** The expression of circEZH2 and linear EZH2 were identified after treated with actinomycin D for 4 h, 8 h, 12 h, 24 h. **i** The expressions of circEZH2 among different BC cells were identified by RT-qPCR. **j** In primary BC tissues (*n* = 20) and liver metastatic tissues (*n* = 11), RT-qPCR was used to verify the expression of circEZH2. The results were analyzed by unpaired Student’s t test. **k** To determine the Kaplan-Meier survival of BC patients (*n* = 115), log-rank tests were used. **l** In BCLM sample, FISH analysis showed circEZH2 overexpression significantly (magnification, X4 scale bar, 200 μm and X20 scale bar, 50 μm). The data was revealed as the mean ± SD and all experiments were repeated at least three times, ns: no significant; * *p* < 0.05; ** *p* < 0.01; *** *p* < 0.001

Three sets of BC primary tumor specimens and paired hepatic metastatic samples were analyzed using circRNA microarrays to discover the circRNAs with differential expression in BCLM. Next, while we define following filter criteria as (1) *P* value < 0.005 (2) fold-change ≥2 (3) recorded in circBase, deepbase and Rybak-Wolf 2015 dataset (4) Gene symbol was metastatic related according to recent research (5) detected and upregulated in clinical BCLM specimens compared to BC primary samples, circEZH2 (hsa_circ_0008324) was identified among all the upregulated circRNAs (Fig. 1a-b). CircEZH2 consisted of three exons (exon 2, exon 3 and exon 4) of EZH2 which were highly conservative in other species such as rhesus, mouse, dog et al. Then Sanger sequencing was conducted to identify back-spliced junction region of circEZH2 which proved the existence of circEZH2 (Fig. 1c). To determine whether circEZH2 was back-spliced, we devised convergent primers as well as divergent primers to amplify the transcription of circEZH2 and linear EZH2 respectively. It was found that unlike linear EZH2, circEZH2 was amplified in cDNA only with divergent primers, whereas linear EZH2 was amplified in both gDNA and cDNA with convergent primers, indicating that circEZH2 was a back-spliced circRNA (Fig. 1d). Afterwards, RNase R assay were conducted to reveal that circEZH2 was more tolerated than its linear EZH2 and GAPDH, which indicated that circEZH2 was circular structure (Fig. 1e). Moreover, actinomycin D test was performed to evaluate the stability of circEZH2, which showed that circEZH2 had the longer metabolic half-life period than linear EZH2 (Fig. 1h). All above suggested that circEZH2 was consistent with the characteristics of back-spliced circRNA that was stable enough to be potential biomarker. After that, we separated and extracted the nuclear and cytoplasmic partitions of BC cells and found that circEZH2 was major situated in the cytoplasm rather than the nucleus via RT-qPCR (Fig. 1f). Fluorescence in situ hybridization (FISH) assays were performed with circEZH2-targeted Cy3 probe to confirm the cytoplasmic location of circEZH2 as well (Fig. 1g). To further identify the clinical meaning of circEZH2, RT-qPCR was performed to find that as compared with normal breast epithelial cells, MCF-10A, circEZH2 was markedly upregulated among BC cell lines, especially in TNBC cell lines (Fig. 1i). Then we performed RT-qPCR in clinical specimens to identify that circEZH2 was upregulated in BCLM specimens comparative with BC primary specimens (Fig. 1j). Moreover, Kaplan-Meier results indicated that upregulated expression of circEZH2 predicted a worse prognosis in BC patients (Fig. 1k). Meanwhile, circEZH2  FISH assays were conducted to show that circEZH2 was upregulated notably in BCLM comparison with its adjacent benign liver tissue (Fig. 1l). As shown above, circEZH2 has been associated with BCLM and poor prognosis in BC.

### CircEZH2 promotes the proliferation and metastasis of BC in vitro

**Fig. 2:**
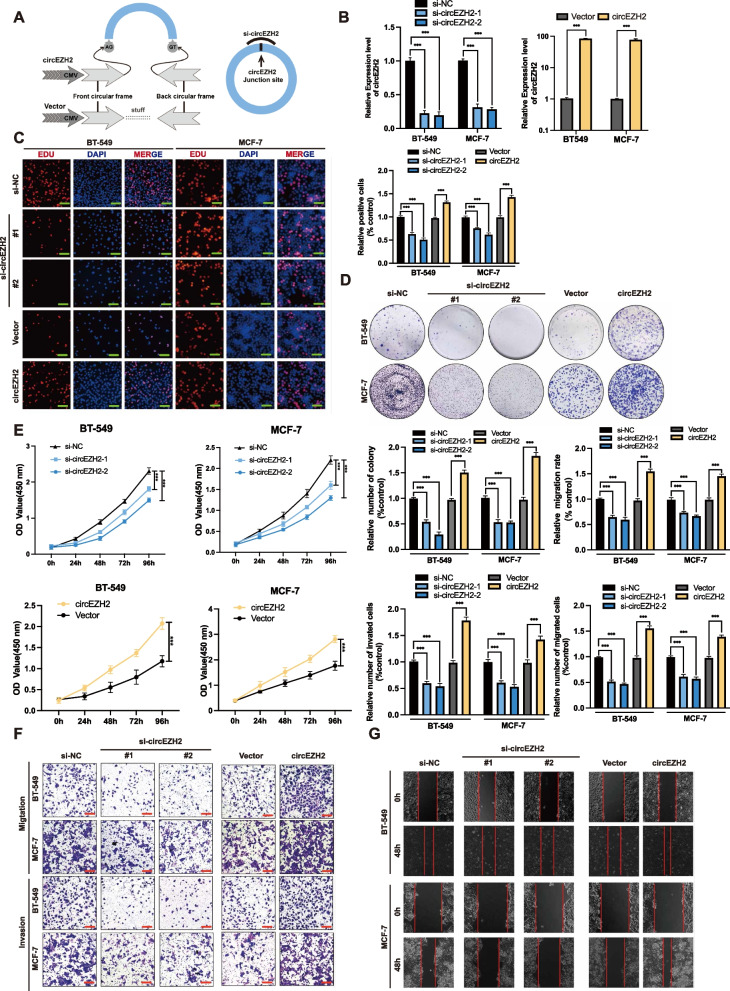
CircEZH2 accentuate vitality, migration, and invasion of BC cells in vitro. **a** Here is a diagram of mechanisms about circEZH2 overexpression with plasmid and knockdown with siRNA. **b** We determined the success of knockdown as well as overexpression of circEZH2 using RT-qPCR. **c-e** To evaluate the vitality in BC cells, EdU assays (Scale bar, 50 μm), colony formation assays and CCK8 assays were conducted. **f** and **g** Migration, invasion of transwell (Scale bar 100 μm) and wound-healing assays (Scale bar 200 μm) were conducted to determine the ability of metastasis in BC cells. The data was revealed as the mean ± SD and all experiments were repeated at least three times, * *p* < 0.05; ** *p* < 0.01; *** *p* < 0.001

In order to investigate circEZH2’s potential functions in BC, we constructed a circEZH2-overexpression plasmid and two small interfering RNAs (siRNAs) (Fig. 2a). Next, we identified that circEZH2 was markedly overexpressed or downregulated in BC cell lines after transfected with circEZH2 overexpression plasmids or siRNAs, respectively (Fig. 2b). After knockdown or overexpression of circEZH2, linear EZH2 transcripts did not significantly change (Additional file 1: Fig. 1a). The EdU assay, CCK-8, and colony formation were used in order to evaluate the vitality ability of BC cells. The viability of BC cells was enhanced by circEZH2 overexpression and notably suppressed by its downregulation (Fig. 2c-e). Then, migration, invasion transwell assays as well as wound-healing experiments were used to find that downregulation of circEZH2 inhibited the migrative and invasive ability in BC cells, whereas overexpression of circEZH2 elicited totally adverse effects (Fig. 2f-g). The results above concluded that circEZH2 promote oncogenesis and metastatic ability of BC cells in vitro.

### CircEZH2 accentuates the development of the xenograft tumors and hepatic metastasis in vivo

**Fig. 3:**
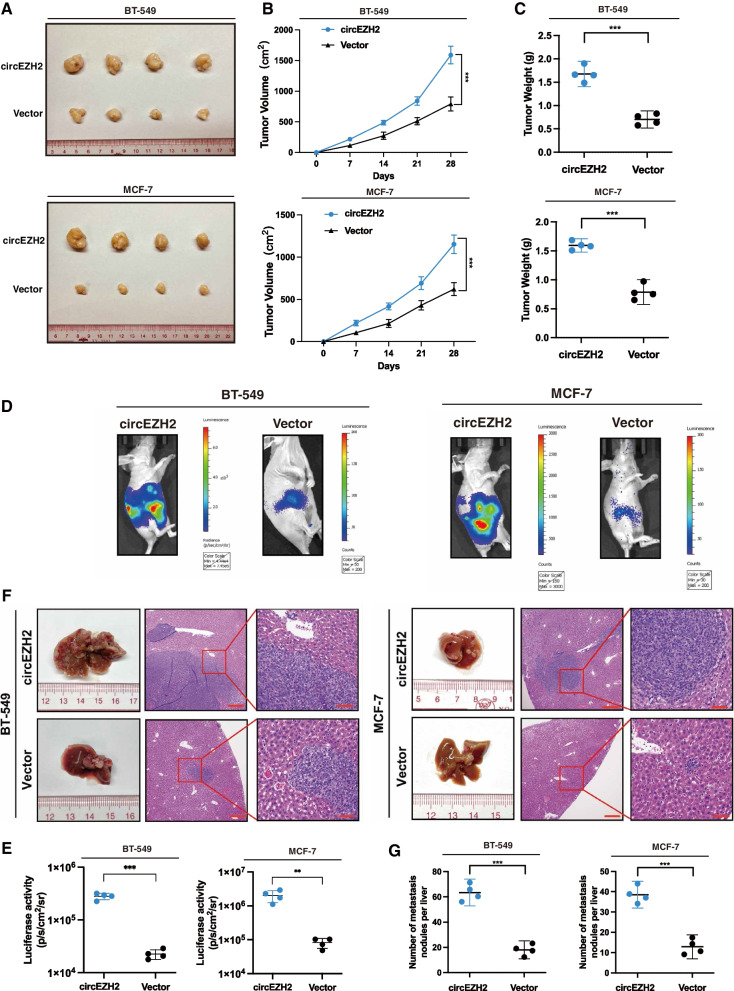
circEZH2 accentuates tumorigenesis and liver metastasis of BC cells in vivo. **a** Pictures of xenograft tumors were shown in the circEZH2 overexpression group and relative control group (*n* = 4). **b** The growth curves of each group of xenograft tumors were displayed. **c** The xenograft tumor weight was measured and analyzed. **d** and **e** Inferior hemi-spleen implantation mice models in vivo were analyzed by IVIS to identify the potential of circEZH2 in promoting liver metastasis. **f** and **g** Metastatic sites in the liver were noted to be increased in the overexpressed circEZH2. On the H&E staining liver slides, metastases were marked with arrows. (Magnification, X4 scale bar 200 μm; X20 scale bar 40 μm). The data was displayed as the mean ± SD and all experiments were repeated at least three times, **P < 0.01, ***P < 0.001

In order to identify the effects of circEZH2 on tumorigenesis of BC tumors in vivo, human-derived BC xenograft nude mice models were constructed via subcutaneously injection by MCF-7 cells and BT-549 cells overexpressing circEZH2 stably, and corresponding control cells in the BALB/C nude mice, while the circEZH2-overexpressed tumors were significantly larger and weighed higher than their relevant control tumors (Fig. 3a-c). To study the effects of circEZH2 in BCLM, MCF-7 and BT-549 with stable circEZH2-overexpressed cells with their control cells were injected into the inferior hemi-spleen to induce BCLM. CircEZH2-overexpressed mice had significantly higher luciferase activity and more liver nodules than the controls (Fig. 3d-g). This all correlated with the results in vitro, indicating that circEZH2 would accentuate BC oncogenesis and hepatic metastatic ability.

### FUS accelerates the back-splicing program of circEZH2

**Fig. 4:**
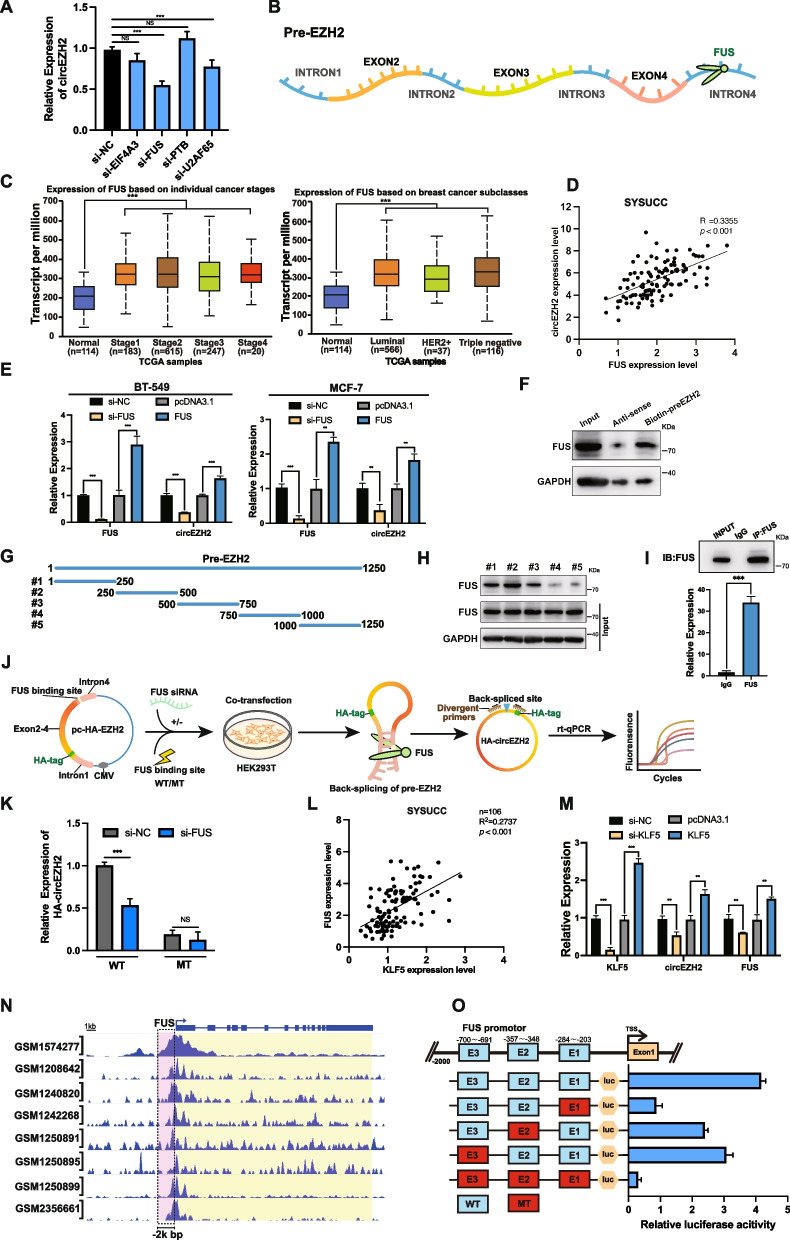
FUS promotes back-splicing program of circEZH2 and KLF5 activates FUS transcription. **a** RT-qPCR was used to determine the expression of circEZH2 after transfected with siRNA targeting EIF4A3, FUS, PTBP1, U2AF65 and control. **b** Schematic diagram showed that FUS binding downstream intron4 region of pre-EZH2**. c** TCGA analysis of FUS expression in different stages and subtypes of BRCA.. **d** Clinical BC samples from SYSUCC were used by RT-qPCR analysis revealing the spearman correlation evaluation between circEZH2 and FUS transcripts. **e** RT-qPCR was performed to reveal the assoicated expression of circEZH2 when FUS was overexpressed or knocked down in BC cells. **f** Western blots were used to determine the resultants of RNA pull-down assay using biotin-preEZH2-probe. **g** Diagram revealing five truncated biotinylated pre-EZH2 probes were constructed. **h** RNA pull down assays were performed by five truncated probes and the resultants analyzed by western-blot were displayed. **i** Lysates of BT-549 were subjected to RIP by anti-FUS antibody and anti-IgG antibody and the enrichments of IP and IgG groups were displayed. **j** Flow illustration showed that firstly pc-HA-EZH2, a novel back-splicing formation validation vector by adding a HA label to the 5′-second EZH2 exon to differentiate the internal circEZH2 was constructed. Secondly, FUS binding region was mutated of pc-HA-EZH2 as mutant type (MT). After that MT and WT vector were transfected into HEK293T with/without knockdown of FUS. Eventually, the back-splicing efficiency of circEZH2 was deternmined by divergent primers across HA tag region and RT-qPCR while linear HA-EZH2 was used as reference. **k** The back-splicing efficiency of circEZH2 was identified by RT-qPCR in WT and MT groups. **l** In SYSUCC clinical human BC tissues, expression of KLF5 was positively correlated with FUS as determined by Spearman’s correlation analysis. **m** RT-qPCR was used to find the associated expression of KLF5, circEZH2 and FUS in change the expression of KLF5. **n** Eight ChIP-seq datasets showed that there were binding peaks within 2k bp upstream promotor of FUS by KLF5. **o** Top, JASPAR predicted potential three binding regions between KLF5 and the promotor of FUS. Bottom, each of three binding sites were mutated in FUS promotor dual-luciferase plasmid while wild type without mutation was positive control and all mutated group was negative control. The results of each group were quantitated by dual-luciferase assays. The data was showed as the mean ± SD and all experiments were repeated at least three times, ns no significance, **P < 0.01, ***P < 0.001

According to circInteractome [19], there were four potential RNA binding proteins on the downstream or upstream introns of pre-EZH2 (which was the immature status of circEZH2 before back-splicing process) which were EIF4A3, FUS, PTBP1 and U2AF65. To further screen these potential regulation candidates, siRNAs targeting each candidate were designed and transfected into BT-549 cells, which revealed that FUS was the best influencer of circEZH2 expression among these candidates (Fig. 4a-b). Therefore, we desired to figure out the regulation mechanism of circEZH2 by FUS. According to the TCGA database, we found that the FUS transcript was upregulated in stage1–4 of BRCA comparison with the normal samples. Meanwhile, FUS expression was upregulated in luminal, HER2+ and TNBC subtypes compared with normal samples, which revealed that FUS might play a significant role in initiation of BC tumorigenesis. (Fig. 4c). Moreover, SYSUCC BC samples were analyzed by RT-qPCR to find that there existed a positive association between the expression of circEZH2 and FUS in mRNA level. (Fig. 4d). Next, RT-qPCR revealed that there existed a direct tendency between FUS and circEZH2 in BC cells when the expression of FUS was altered (Fig. 4e). In our hypothesis, FUS might promote the process of back-splicing of circEZH2 via binding with the 3′-flanking-intron of circEZH2. To further validate our conjecture, we constructed biotinylated pre-EZH2 probe containing 1250 bp downstream of EZH2 exon 4. Next, RNA pull-down assays were performed to find that pre-EZH2 probe enriched FUS successfully compared with its control group (Fig. 4f). However, intending to localize the precise binding site of FUS on pre-EZH2, five truncated biotinylated probes of 1250 bp pre-EZH2 full-length probe were constructed, while RNA pull-down identified most FUS-enriched part was the second truncated biotinylated probe of pre-EZH2 (Fig. 4g-h). Afterwards, we performed RNA immunoprecipitation (RIP) to validate the interplay between pre-EZH2 and FUS protein (Fig. 4i). To further confirm that FUS acted as an indispensable role in the program of circEZH2 back-splicing, we designed the pc-HA-EZH2 vector to verify the back-splicing efficiency of circEZH2 under influence of FUS. Besides, the unvaried pc-HA-EZH2 as wild type (WT) while the one of which the FUS binding site sequences were mutated was mutated group (MT). After transfected pc-HA-EZH2 WT or MT into HEK-293 T which had been transfected with FUS-targeted siRNA, the results showed that the back-splicing efficiency of circEZH2 was decreased significantly when knockdown the expression of FUS or mutation of FUS binding site of pc-HA-EZH2 (Fig. 4j-k). In sum, the above suggests that FUS could promote back-splicing process of circEZH2 by binding to the 3′-flanking intron part of pre-EZH2.

### CircEZH2 adsorbs miR-217-5p to inhibit its function

**Fig. 5:**
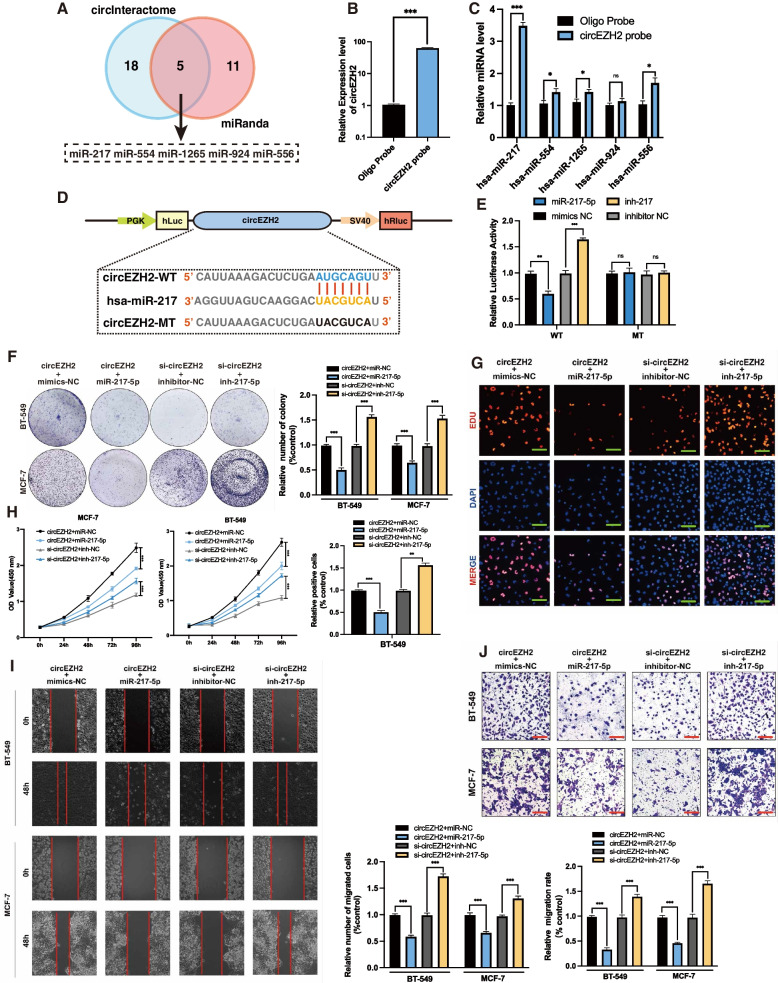
CircEZH2 adsorbs miR-217-5p. **a** CircEZH2 exhibits potential binding regions for miRNAs in the intersection between CircInteractome and miRanda databases, as indicated on the Venn diagram. The illustration revealed the potential binding miRNAs with circEZH2. **b-c** Right, CircEZH2 RNA pull down assay was performed to find that hsa-miR-217-5p was the most enriched one. Left, the biotin-miR-217-5p probe could enrich circEZH2 effectively. **d** and **e** Left, the diagram states the dual-luciferase plasmids with mutant (MT) and wild (WT) containing binding regions of miR-217-5p**.** Right, the relative luciferase activities were determined after co-transfection of circEZH2-MT or circEZH2-WT and miR-217-5p inhibitors or mimics with control respectively. **f-h** Co-transfections of BC cells with indicated vector, siRNA, miRNA, or inhibitor were performed to validate their viability by colony formation assay, EdU assay (Scale bar, 50 μm), and CCK8 assay, respectively. **i** and **j** The migration ability in BC cells co-transfected with described vector, siRNA, miRNA or inhibitor was analyzed by migration transwell (Scale bar 100 μm), and wound healing assay (Scale bar 200 μm), respectively. The data was displayed as the mean ± SD and all experiments were repeated at least three times, ns no significant, **P < 0.01, ***P < 0.001

To further identify biological mechanism of circEZH2, bioinformatic analysis based on miRanda [20] and circInteractome [19] was conducted to find the potential binding regions between miRNAs and circEZH2. The results showed that there were five miRNAs in the intersection which were miR-217, miR-554, miR-924 and miR-556 (Fig. 5a). Then, circEZH2-RNA pull-down assay was performed to reveal that miR-217-5p was most enriched one in candidates above (Fig. 5b-c). According to TCGA, 1165 pairs of tissues were analyzed, with BRCA tissues expressing significantly less miR-217-5p than the normal breast tissues (Additional file 1: Fig. 1c), which revealed that miR-217-5p could play a role as a potential tumor suppressor. To further validate the interplay between miR-217-5p and circEZH2, wild-type dual-luciferase vector including circEZH2 sequences and mutant-type vector containing predicted binding regions between circEZH2 and miR-217-5p were established (Fig. 5d). The results revealed that miR-217-5p inhibitors significantly promoted luciferase activity, while a notable inhibition of luciferase activity was observed after treatment with miR-217-5p mimics, whereas no significant relative luciferase activity change was detected in mutant-type (Fig. 5e), which proved that circEZH2 directly adsorbed miR-217-5p. In vitro study of the function interplay of cirEZH2 and miR-217-5p, rescue experiments via co-transfecting miR-217-5p inhibitor or mimic with si-circEZH2 or circEZH2 overexpression vector, respectively, into BC cells were performed. The results of EdU, CCK-8, transwell of migration assay with wound healing assay revealed that miR-217-5p mimics reversed the oncogenic tendency elicited via overexpression of circEZH2 while inhibition of miR-217-5p vividly changeover the phenotypes provoked by knockdown of circEZH2 in vitro (Fig. 5f-j). In summary, circEZH2 could adsorb with miR-217-5p and turnover its cancer suppression function, thereby accentuating tumorigenesis and progression in BC.

### KLF5 is the distinct target regulated by circEZH2/miR-217-5p axis to promote metastasis

**Fig. 6:**
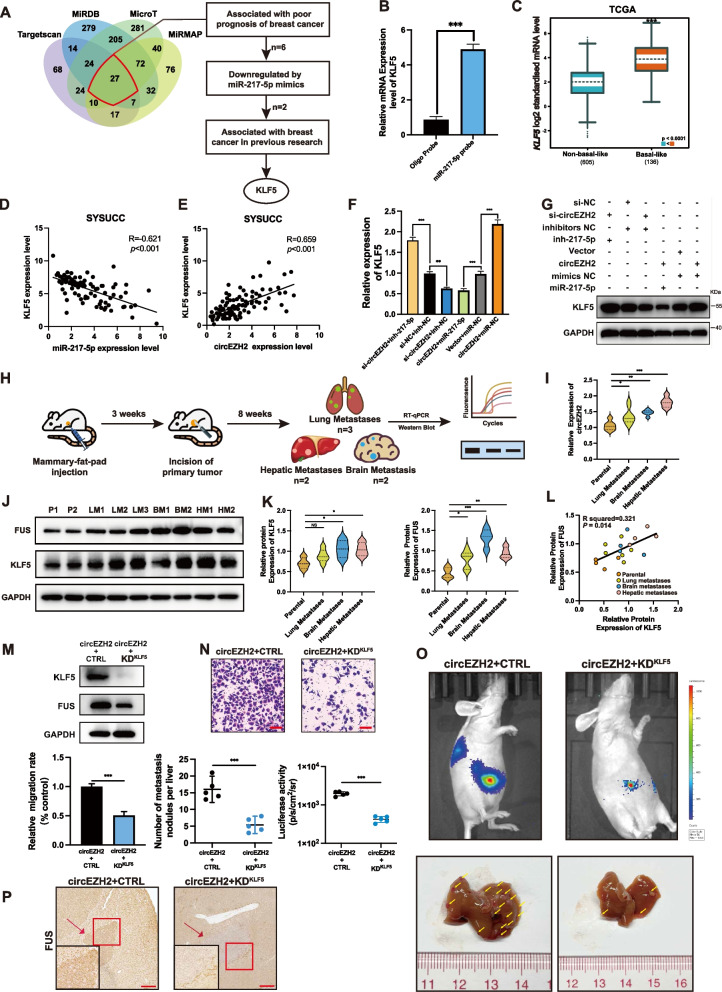
KLF5 is an distinct target of miR-217-5p. **a** Left, The Venn diagram revealed the potential targeted gene of miR-217-5p among miRDB, microT, Targetscan, and miRMAP four databases; Right, the flow chart to filter for target gene of miR-217-5p step by step.. **b** RNA pull down assays were performed by biotin-miR-217-5p-probe to determine enrichment of KLF5 through RT-qPCR analysis. **c** Through TCGA analysis by bc-GenExMiner, the KLF5 expression was upregulated in basal-like subtype of BC **d** and **e** Right, miR-217-5p expression was negative correlated with KLF5 in BC samples in transcript analysis of clinical BC samples from SYSUCC. Left, the expression of circEZH2 was positive correlated with KLF5 in clinical BC tissues from SYSUCC in mRNA level. Both were conducted by spearman correlation analysis. **f** and **g** RT-qPCR, and western blots were used to analyze KLF5 expression after co-transfection with siRNA, vector, mimic or inhibitor as indicated. **h** Flow diagram indicated that establishment of xenograft-induced metastatic mice model and multi-organ metastases were analyzed by western blot and rt-qPCR. **I** RT-qPCR was conducted to reveal the expression pattern in different metastases compared with parental cells. **j** and **k** The expression FUS and KLF5 in different metastases were determined by western blot. P = parental; LM = lung metastases; BM = brain metastases; HM = hepatic metastases. **l** The pearson correlation analysis was performed to identify the protein expression association between FUS and KLF5 in metastases. **m** The doxycycline-induced knockdown of KLF5 in circEZH2 overexpresed cells was verified by westernblot. **n** and **o** Migration Transwell rescue assay and liver metastatic mice rescue models were performed to prove the influence of KLF5 knockdown on circEZH2 overexpression cells. The scale bar of Transwell was 100µm. The liver metastases were quantitated by IVIS and number of liver metastases were marked with yellow arrows. **p** Immunohistochemistry (IHC) of FUS was performed in metastases of KLF5 knockdown group and its control group (Low power field: Scale bar 100 μm; High power field: Scale bar 20µm). The data was showed as the mean ± SD and all experiments were repeated at least three times, ns no significant, *P < 0.05 **P < 0.01, ***P < 0.001

In order to uncover the deep interplay mechanism by which circEZH2/miR-217-5p axis worked, we performed bioinformatics analysis among miRMap [21], microT [22], miRDB [23], TargetScan [24] databases according to each threshold of binding scores and found that there were 27 genes in the intersection among four databases. After that, a more precise algorithm was used to bolt these candidates: Firstly, KM-plot analysis in TCGA was conducted to find candidate genes associated with BC poor prognosis; Secondly, RT-qPCR was performed to define the downregulated genes with miR-217-5p mimics; Thirdly, we searched previous researches to locate KLF5 as our final target of miR-217-5p which was associated with BC poor prognosis and had been reported as important oncogenic transcription in BC [25, 26]. (Fig. 6a). To further validate the binding relation between miR-217-5p and KLF5, RNA pull down assay was performed by biotin-miR-217-5p probe to find that miR-217-5p could enriched the 3′ untranslated region (UTR) of KLF5 compared with control probe (Fig. 6b). Moreover, clinical BC specimens from SYSUCC were analyzed by RT-qPCR to find that the expression of KLF5 was negatively correlated with miR-217-5p (Fig. 6d). The dual-luciferase assay intending to prove the post-transcription regulation between KLF5 and miR-217-5p had been proved in our previous research [18] The results above identified that KLF5 was downregulated by miR-217-5p. Meanwhile, it was urgent to define the relation between circEZH2 and KLF5, on the one hand, clinical BC samples from SYSUCC were used to reveal a positive correlation between circEZH2 and KLF5 (Fig. 6e), on the other hand, while circEZH2 was overexpressed or downregulated, RT-qPCR and western blot were taken to find that circEZH2 could upregulate mRNA and protein of KLF5 (Additional file 1: Fig. 1e-f). After that, miR-217-5p inhibitor were transfected into the circEZH2-knockdown group and transfection of miR-217-5p mimics into circEZH2-overexpression groups were used as rescue tests to reveal that miR-217-5p mimic significantly reduced the increase in KLF5 expression in the circEZH2-overexpressed group, and KLF5 expression was significantly restored by miR-217-5p inhibitor when circEZH2 was knocked down (Fig. 6f-g; Additional file 1: Fig. 1i). The results above identified that KLF5 was distinctly upregulated by circEZH2/miR-217-5p axis. Next, according to TCGA KM-Plotter [27] proved that KLF5 overexpression was associated with worse relapse-free survival in BC patients (Additional file 1: Fig. 1h) and upregulated in basal-like BC subtype analyzed by BC GenExMiner [28] (Fig. 6c). Meanwhile, immunofluorescence (IF) of BCLM specimens from patients were performed and the results revealed that KLF5 was notably increased in liver metastases than in normal hepatic tissues (Additional file 1: Fig. 1e). All of them identified that KLF5 expression had a positive relationship with BC poor prognosis and liver metastasis. However, considering multi-organ metastatic situation of BC and heterogeneity of cancer cells, we established xenograft-induced metastasis mice models by BT-549 cells in balb/c nude mice. In the first, we injected BT-549 cells into mammary pad of 5 mice to promote xenograft tumor growth. Then, we performed the tumorectomy when the tumor short diameter approached 2 cm. After 8 weeks, it was found that 2 mice had hepatic metastasis and brain metastasis while 3 mice had lung metastasis (Fig. 6h). Metastases of each mouse were resected and extracted protein and RNA to perform western blot and RT-qPCR compared with parental cells. The results showed that circEZH2 was upregulated in hepatic metastasis, brain metastasis and lung metastasis, of which circEZH2 was most upregulated in hepatic metastases (Fig. 6i). In the meantime, KLF5 was upregulated in hepatic and brain metastasis while FUS was increased in hepatic, brain and lung metastases compared with parental cells in protein level (Fig. 6j-k). Moreover, the protein expression of KLF5 and FUS was measured to be positive correlated which conformed to our results in clinical BC specimens (Fig. 6l). The xenograft-induced metastatic mice model revealed that circEZH2, KLF5 and FUS were upregulated in hepatic metastases as well as metastases of brain or lung, which helped us opened a vision that FUS/circEZH2/KLF5 might provoke multi-metastasis mechanism. In order to identify the liver metastatic regulation of circEZH2 and KLF5 in vivo, we established doxycycline induced KLF5-shRNA cell lines under circEZH2 overexpression by pLKO-Tet-On-shRNA plasmid (Addgene, Plasmid #98398). After that, doxycycline-induced knockdown of KLF5 was verified by western blot and the expression of FUS was downregulated after knockdown of KLF5 (Fig. 6m). Then migration transwell and liver metastatic models were performed by this cell line with/without doxycycline. The results showed that KLF5 knockdown vividly reversed the migration ability elicited by circEZH2 overexpression in vitro and the metastatic nodules and luciferase activity was significantly decreased after knockdown of KLF5 in the baseline of circEZH2 overexpression (Fig. 6n-o). Meanwhile, IHC was performed to reveal that FUS was decreased in hepatic metastases in KLF5 knockdown group compared with control group (Fig. 6p). All above proved that not only KLF5 inhibition could downregulate the expression of FUS, but also KLF5 was essential for circEZH2 to promote liver metastasis in vitro and vivo.

### KLF5 enhances the transcription of FUS and a novel positive feedback loop circEZH2/KLF5/FUS is established

According to the RT-qPCR analysis of clinical BC samples from SYSUCC, FUS expression had notably positive correlation with KLF5 in mRNA level (Fig. 4l). When we increased or decreased the expression of KLF5, the expressions of FUS and circEZH2 were vividly upregulated and downregulated respectively (Fig. 4m), whereas there was no significant change of the linear EZH2 (Additional file 1: Fig. 1b), which inspired us that KLF5 might upregulate FUS in transcriptional level. Afterwards, we analyzed 8 ChIP-seq datasets by Cistrome data browser [29] to find that there were KLF5 enriched peaks in 2k bp of FUS promotors (Fig. 4n). According to JASPAR [30], three KLF5 binding regions were predicted on the promoter of FUS. In order to test whether KLF5 enhanced transcription of FUS, Chromatin Immunoprecipitation (ChIP) assays were performed to find that among the three possible binding regions for KLF5 (E1, E2, E3), the E1 region (− 284 ~ − 203) was the best enriched one which was consistent with our previous research results (Additional file 1: Fig. 1g). Nevertheless, to further confirm the interplay between KLF5 and three binding sites of the promotor of FUS, promoter dual-luciferase assays were performed in mutation of each binding site respectively. Meanwhile, non-mutated group was used as positive control while all-site-mutated group was considered as negative control. The dual-luciferase results showed that E1 region was the most essential for KLF5-induced transcription activation revealing that KLF5 enhanced FUS transcription to increase circEZH2 through accentuating its backs-splicing program (Fig. 4o). All the above concluded that FUS/circEZH2/KLF5 built a novel positive feedback loop.

### circEZH2 promotes EMT of BC in CXCR4-induced way via KLF5

**Fig. 7:**
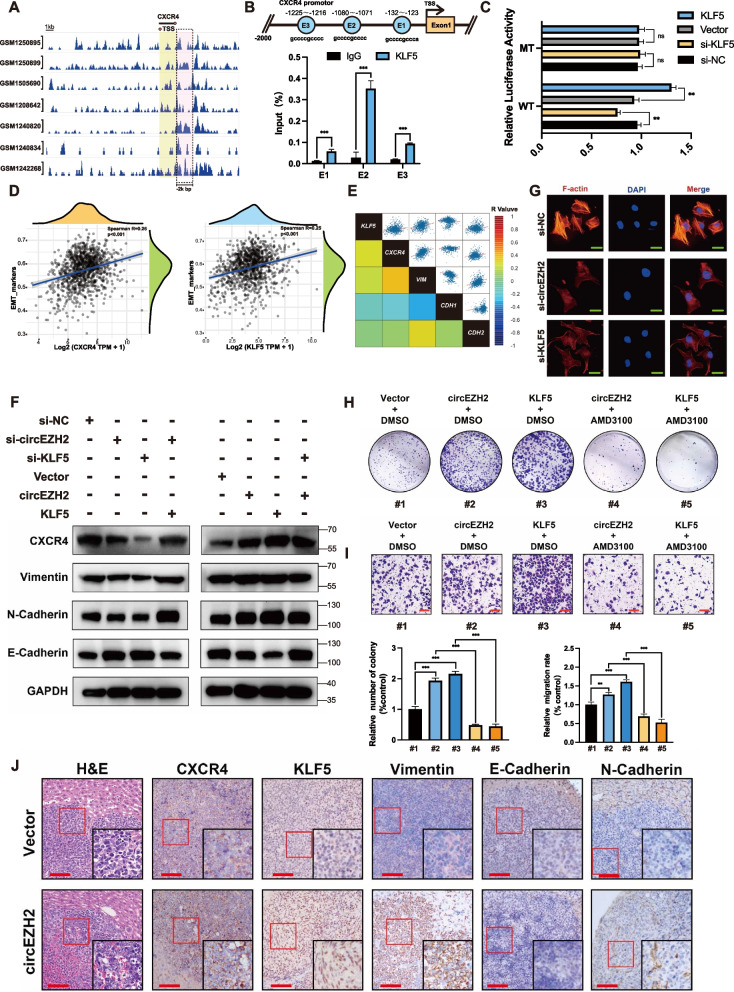
FUS/circEZH2/KLF5 loop promotes CXCR4-induced EMT program. **a** Seven ChIP datasets showed that there were KLF5 peaks within 2k bp promotor of CXCR4. **b** Top, the potential binding sites on the promotor of CXCR4 by KLF5 according to JASPAR. **c** Dual luciferase assays were conducted in FUS promotor wild type group and E2 mutated group. **d** According to TCGA GSVA analysis, KLF5 and CXCR4 were positive correlated with EMT markers respectively **e** Based on STARBASE database, regression correlation rates among KLF5, CXCR4, VIM, CDH1 and CDH2 transcripts were displayed in heatmap. **f** Western blot was conducted to reveal the change of CXCR4, Vimentin, N-cadherin and E-cadherin after indicated siRNA or Vector. **g** IF was conducted to find that F-actin were vividly downregulated after knockdown circEZH2 and KLF5 respectively. **h **and** i** The CXCR4-rescue colony formation assay and CXCR4-rescue migration Transwell assay were conducted within indicated Vector or drug (Scale bar 100 μm) **j** IHC were performed in circEZH2 overexpressed group of BCLM nude mice and control group with indicated antibody (Low power field: Scale bar 100 μm; High power field: Scale bar 20µm. ). The data are showed as the mean ± SD and all experiments were repeated at least three times, ns no significant, *P < 0.05 **P < 0.01, ***P < 0.001

**Fig. 8:**
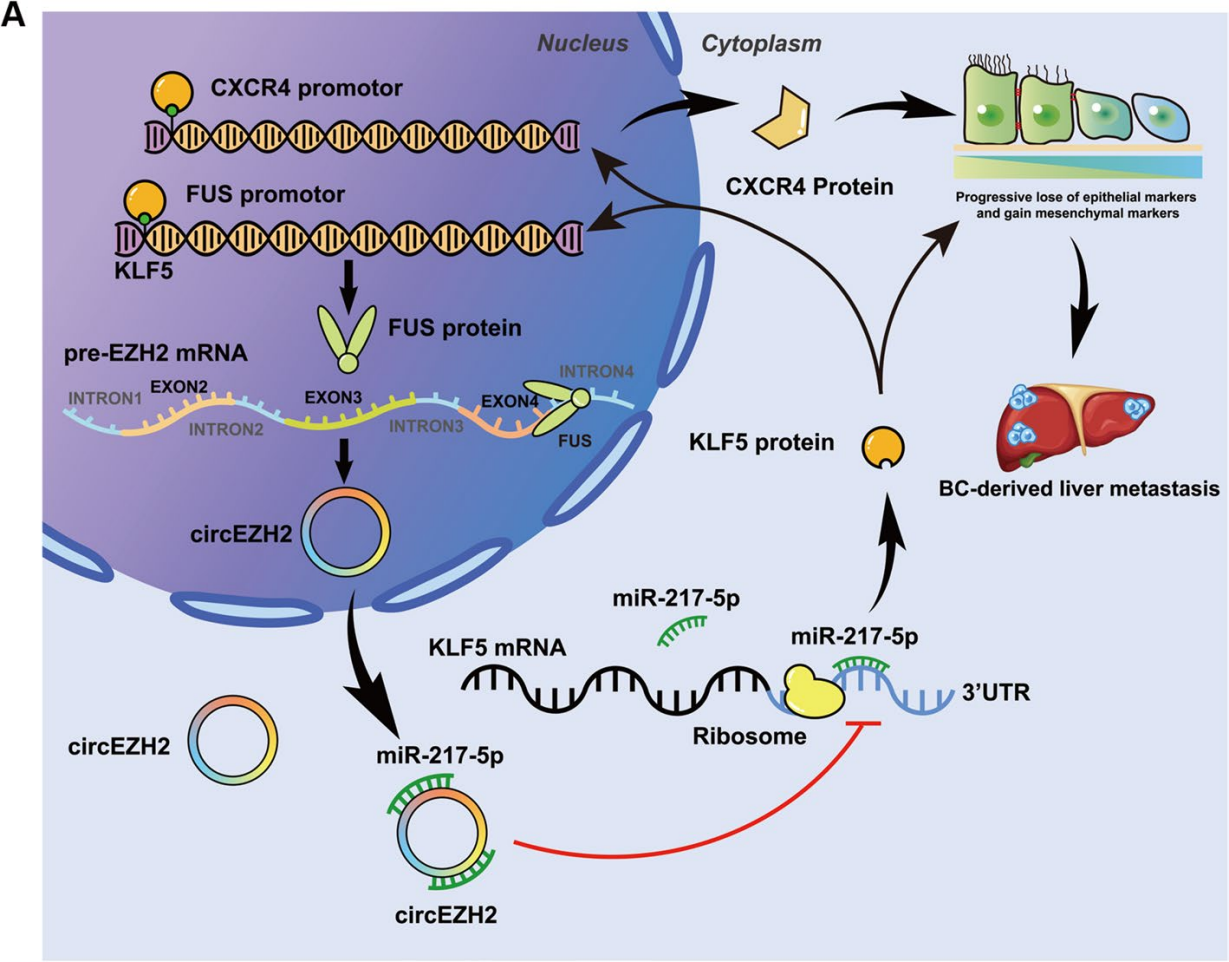
**a** The diagram explains the biological mechanism of a novel positive feedback loop FUS/circEZH2/KLF5 through which liver metastasis of breast cancer was induced by enhancing epithelial-mesenchymal transition (EMT) via up-regulation of CXCR4

According to the STARBASE, we found that KLF5 was positive correlated with CXCR4 in RNA expression (Additional file 1: Fig. 1j). CXCR4 was relative with poor prognosis in BRCA patients and upregulated in primary BRCA tumors compared with normal samples (Additional file 1: Fig. 1k-m). We analyzed seven ChIP-seq datasets by Cistrome data browser [29] to find that there were potential KLF5 binding peaks in 2kbp upstream of CXCR4 TSS site (Fig. 7a). Next, we predicted the potential binding regions of KLF5 on CXCR4 promotor via JASPAR [30]. The results revealed that there were three binding regions named E1, E2, and E3. Then, ChIP assay was conducted to demonstrate that KLF5 could bind with these potential sites while E2 region was the most enriched with KLF5 (Fig. 7b). Therefore, we conducted promoter dual luciferase assays to identify that relative luciferase activity was correlated with KLF5 expression in wild-group while there existed no notably change in mutated group (Fig. 7c). All above validated that KLF5 could bind on CXCR4 promotor to activate its transcription. To discover the relations among KLF5, CXCR4 and epithelial mesenchymal transition (EMT), STARBASE [31] was used to define the correlation among KLF5, CXCR4 and EMT markers and these correlation rates were displayed as heat-map according to R value respectively. The results identified that the expression of EMT markers were positive associated with KLF5 and CXCR4 while the spearman R of KLF5 and CXCR4 was extremely close (Fig. 7d). With deeper correlation analyses, we found that KLF5 and CXCR4 was positively relative with Vimentin and N-cadherin while they were negative significantly with E-cadherin while the coefficients of KLF5 or CXCR4 among these EMT markers were extremely similar (Fig. 7e), which inspired us whether KLF5 upregulated by circEZH2 promoted EMT of BC in CXCR4 induced way. Therefore, western blots were performed to validate that circEZH2 overexpression accelerated EMT of BC and increased CXCR4 expression while knockdown KLF5 in circEZH2-overexpressed group the EMT and CXCR4 expression were literally reversed (Fig. 7f). Meanwhile, we performed F-actin IF assay to find that the expression of F-actin was decreased after knockdown circEZH2 or KLF5. All the results above demonstrate that circEZH2 could promoted EMT of BC in CXCR4-induced way via upregulated KLF5. To further demonstrate the function induced by CXCR4, we took CXCR4 inhibitor AMD3100 to perform colony formation assays as well as transwell assays. AMD3100 has demonstrated a vivid ability to inhibit proliferation and migration elicited by circEZH2 or KLF5, which indicated CXCR4 played an important role in FUS/circEZH2/KLF5 positive feedback loop and AMD3100 might be a potential target to inhibit this oncogenesis loop (Fig. 7h-i). To further discover the relations between EMT and circEZH2, hepatic metastases of nude mice were taken to conduct IHC. We found that KLF5, CXCR4 vimentin and N-cadherin were upregulated in circEZH2 group while E-cadherin were downregulated (Fig. 7j), which demonstrated that FUS/circEZH2/KLF5 feedback loop could promoted EMT of BC in CXCR4-induced way thus leading to BCLM (Fig. 8a).

## Methods

### Patient specimens

The BCLM samples and primary BC specimens were assembled from BC patients from Sun Yat-Sen University Cancer Center (SYSUCC) in Guangzhou, China which were conducted by RNA later instantly after gathered. We obtained all samples from patients with informed consent under institutional review board consented protocols.

### Cell lines and culture conditions

There are six human BC cell lines, MDA-MB-231, BT-549, T-47D, MCF-7, and SKBR-3 and the breast normal epithelial cell line, MCF-10A with HEK293T used in this study (ATCC, Manassas, VA, USA). A selection of cell lines, including MDA-MB-231, SKBR-3, BT-549, T-47D, MCF-7, HEK293T, and SKBR-3, were all grown in DMEM medium (Gibco, USA), while MCF-10A cells were grown in DMEM/F-12 medium (Gibco, NY, USA). The media mentioned above were replenished with 10% fetal bovine serum (Gibco, NY, USA) and 50 U/ml streptomycin and penicillin (Gibco, NY, USA). This study used a humidified incubator (Thermo Fisher Scientific, MA, USA) to culture all the cells at 37 °C in 5% CO2.

### Animal experiments

Female BALB/C nude mice were obtained from Vital River Laboratory (Beijing, China) and fostered in the Center for Experimental Animals of Sun Yat-Sen University under standard conditions. For xenograft proliferation mice models, we subcutaneously injected MCF-7 and BT-549 cells (2 × 10^6^ in PBS) with stably overexpressed circEZH2 or control cells into the mice that were sacrificed 4 weeks after injection. The volume of tumors was measured weekly and analyzed in line with the standard formula: volume = length x width^2^ / 2, while the weight of tumors was measured after the sacrifice of the mice. In the mice liver metastasis experiments, 1 × 10^6^ MCF-7 or BT-549 cells overexpressing circEZH2 steadily, as well as luciferase, were given injection into the spleen of four- to five-week-old BALB/c female mice, and control cells were given same injection meanwhile. After 60 days, we performed bioluminescence by injecting 100 μl 15 μg/μl D-luciferin intraperitoneally into the mice and captured images by Xenogen IVIS Lumina Series II and processed the results via Living Image 2.11 software (Xenogen Corp.). After that, livers of the mice were removed as soon as they were sacrificed for further pathological research. For xenograft-induced metastatic mice model, firstly, BT-549 cells (2 × 10^6^ in PBS) were injectied into mammary pad of BALB/C nude mice. Secondly, when the short diameter approach 2 cm, tumorectomy was performed to remove the xenograft tumor. After 8 weeks, bioluminescence was performed to observe the status of metastasis. Once metastases were noticed, the mice were sacrificed to separate the multi-organ metastases to extract RNA and protein for further analysis. Sun Yat-Sen University’s Animal Care and Use Committee has approved all the procedures above.

### Statistical analysis

SPSS 21.0 (IBM, IL, USA) and GraphPad Prism 9.1 (GraphPad Inc., CA, USA) were used for statistical analysis. For detecting the significances between groups, we used the student’s t test, while the Spearman correlation coefficient was used to determine the correlation between groups. The survival analysis was conducted by Kaplan-Meier method, while the statistical significance was determined by the log-rank test.

## Discussions

Metastasis is the major cause of BC-related death globally [32]. And the unique biological structure and tissue-specific distribution of circRNAs have recently attracted much attention in tumorigenesis research, which could be a new cancer-related biomarker and potential therapy opportunity for BC [17, 33]. Consequently, it is imperative to validate the biological functional circRNAs to define their mechanism of affecting BC metastasis. In this article, circEZH2 was identified in three paired primary BC samples and BCLM samples via microarray due to its significant upregulation in BCLM samples and correlation with worse prognosis in BC patients. Additionally, we discovered that overexpression of circEZH2 promoted the vitality of cells, invasion of cells, and hepatic metastasis in vivo.

Research has shown that a variety of regulatory factors are involved in the biogenesis of circRNAs from their precursor mRNA, like transcription factor Twist1 [34], variable splicing factor QKI [35], EIF4A3 [36, 37], and super-enhancer YY1 [38]. Moreover, it has recently been identified that FUS was responsible for the biological process of circRNA synthesis [39, 40]. Han et al., for instance, found that FUS could help to promote the metastatic spread of colorectal cancer by back-splicing the circLONP [41]. Our previous research validated FUS could bind precursor of circROBO1 to help its back-splicing process [18]. Meanwhile, in this article, mutation of FUS binding site on pc-HA-EZH2 was performed with intervention the expression of FUS to identify that FUS coud vividly promote back-splicing process of circEZH2 which was not included in our previous research. Moreover, xenograft-induced metastatic mice models have proved that FUS was significantly upregulated in multi-organ metastasis such as hepatic, brain and lung metastasis which implied that FUS might associated with multi-organ process. Consequently, FUS might be a new tumor indicator in BC and might accelerate metastasis by upregulating circEZH2. However, TCGA bioinformatic analysis of FUS was found that FUS was not differentially expressed in different stages of BC but vividly upregulated in BC samples which might implied FUS played an important role in initiation of BRCA. Therefore, it is necessary to identify the deep oncogenesis functions of FUS with further experiments.

The function of circRNAs is highly related with its location within the cell. In our article, circEZH2 was proved to be distributed mostly in the cytoplasm and bioinformatic analysis, RNA pull-down assay showed that circEZH2 could bind to miRNA hsa-miR-217-5p. Various types of cancer have been validated to contain miR-217-5p as an anti-tumor miRNA [42–45]. In our study, we identified that there existed phenotypic interplay between miR-217-5p and circEZH2 while miR-217-5p ectopic overexpression notably remitted the cancerogenic effects elicited by circEZH2, which showed that the biological function of miR-217-5p were regulated via circEZH2.

Known transcription factor KLF5, it has been found to be overexpressed in basal-subtype BC [46]. In BC, KLF5 has been identified as a marker for worse prognosis [47, 48]. Meanwhile, KLF5 accentuated BC progression via upregulating Slug [49], Cyclin D1 [50], Nanog [51], FGFBP1 [52] and TNFAIP2 [25]. Besides, cancer-related metastasis may be facilitated by KLF5 in many cancer types [26, 53, 54]. By using a bioinformatic analysis and RNA pull down assays, we determined that KLF5 was an intimate target for miR-217-5p. Positive correlation was found between KLF5 mRNA and protein expression and circEZH2 expression which could be remitted by ectopic miR-217-5p. Besides, our research proved that the expression of KLF5 upregulated by circEZH2 and oncogenesis of circEZH2 could be remitted by ectopic miR-217-5p. Meanwhile, we performed xenograft-induced metastatic models to prove that KLF5 was upregulated in hepatic and brain metastases, and there existed a positive protein expression correlation between FUS and KLF5. Besides, doxycycline-induced KLF5 knockdown cells were used for transwell assays in vitro and liver metastatic models in vivo which revealed that KLF5 knockdown could vividly decrease the metastatic tendency of circEZH2 overexpression. KLF5 as an effective transcription factor can identify GCCCGCCC motif in the promotors of genes. We predicted KLF5 binding sites in FUS promotor region via ChIP-seq and JASPAR. Afterward, ChIP and promotoer mutated dual-luciferase assay were performed to validate that KLF5 was able to bind on E1 region of FUS promotor and activate its transcriptional program, thus leading to upregulating the expression of circEZH2. Therefore, FUS/circEZH2/KLF5 a positive feedback loop was established.

While BC primary tumors are cured via surgical resection and systematic chemotherapy, cancer with metastasis is mostly incurable or untreatable because currently available therapeutic drugs are ineffective against disseminated BC tumor cells [55]. However, invasion-metastasis cascade is a complicated multi-step process, of which EMT program is a key step in the initiation of metastasis such as detachment, migration, and invasion [56–58]. Zhang et al. found that KLF5 sustained EMT state and oncogenesis to elicit bone metastasis and chemotherapy-resistance in prostate cancer via upregulated CXCR4 [59]. CXCR4 which is G-protein-coupled receptor for the ligand CXCL12 promoted cellular trafficking and migration. Meanwhile, organs such as liver, bone, lung or lymph nodes were enriched with high level of CXCL12, which established CXCR4/CXCL12 axis to mediate BC progression and metastasis [60, 61]. Besides, CXCR4-CXCL12 axis and phosphorylated mTOR could induce EMT program in metastatic BC [62]. Therefore, we defined the relation between FUS/circEZH2/KLF5 feedback loop and EMT program by bioinformatic analyses and western blot, which revealed that FUS/circEZH2/KLF5 could activate transcription of CXCR4 to induce EMT program of BC. Correia et al. found that activated hepatic stellate cells (aHSCs)-secreted CXCL12 which mediated by disseminating tumor cells (DTC) could induce NK cells quiescence thus leading to cancer metastasis [63]. This inspired us that the immune microenvironment in hepatic DTC niche should be taken into deeper research looking for further functions of CXCR4 upregulated in FUS/circEZH2/KLF5 loop. Meanwhile, our previous research proved that KLF5 could inhibit selective autophagy of Afadin, a skeletal protein interacting with claudin-2 which were essential for BCLM [18]. This article proved functions of KLF5 inducing BCLM in the point of EMT which was supplement and continued research of our previous work. Afterwards, CXCR4 would lead us to identify the influence of KLF5 on BCLM into immune microenvironment level.

## Conclusions

A novel circRNA named circEZH2 was identified to promote BC oncogenesis and liver metastasis in BC while BCLM exhibited an upregulation of circEZH2 which correlated with worse prognosis. Furthermore, we found that circEZH2 could reverse KLF5 post-transcriptional inhibition by sponging miR-217-5p which could accelerate CXCR4-induced EMT of BC. Moreover, FUS which was able to help back-splicing process of circEZH2 could be upregulated transcriptionally via KLF5 thus leading to FUS/circEZH2/KLF5/CXCR4 positive feedback loop, which potentially serving as a biomarker for BC metastasis and a target for biological BC therapy.

## Supplementary Information

**Additional file 1.** Supplemental Figure 1

**Additional file 2: Table S1.** Sequences of siRNAs used in this study

**Additional file 3: Table S2.** Primer sequences used in RT-qPCR and PCR analysis

**Additional file 4.** Supplemental Method

## Data Availability

The corresponding authors provided the data used and analyzed in this article upon request.

## Abbreviations

BC: Breast cancer
BCLM: Breast cancer liver metastasis
circRNA: circular RNA
miRNA: micro RNA
DTC: Disseminating tumor cells
TCGA: The Cancer Genome Atlas
siRNAs: Small interfering RNAs
IF: Immunofluorescence
ChIP: Chromatin Immunoprecipitation
EMT: Ephelial mesenchymal transition
KLF5: Krueppel-like factor 5
FUS: RNA-binding protein FUS
CXCR4: C-X-C chemokine receptor type 4
EZH2: Enhancer of zeste homolog 2
PBS: Phosphate-buffered saline
CXCL12: C-X-C chemokine ligand type 12
UTR: Untranslated region
EIF4A3: Eukaryotic translation initiation factor 4A3
PTBP1: Polypyrimidine tract binding protein 1
U2AF65: U2 small nuclear RNA auxiliary factor 2

## References

[1] Siegel RL, Miller KD, Jemal A (2020). Cancer statistics, 2020. CA Cancer J Clin.

[2] He Z-Y, Wu S-G, Peng F, Zhang Q, Luo Y, Chen M (2017). Up-regulation of RFC3 promotes triple negative breast Cancer metastasis and is associated with poor prognosis via EMT. Transl Oncol.

[3] Adam R, Aloia T, Krissat J, Bralet M-P, Paule B, Giacchetti S (2006). Is liver resection justified for patients with hepatic metastases from breast cancer?. Ann Surg.

[4] Pivot X, Asmar L, Hortobagyi GN, Theriault R, Pastorini F, Buzdar A (2000). A retrospective study of first indicators of breast cancer recurrence. Oncology..

[5] Leung AM, Vu HN, Nguyen K-A, Thacker LR, Bear HD (2010). Effects of surgical excision on survival of patients with stage IV breast cancer. J Surg Res.

[6] Eng LG, Dawood S, Sopik V, Haaland B, Tan PS, Bhoo-Pathy N (2016). Ten-year survival in women with primary stage IV breast cancer. Breast Cancer Res Treat.

[7] Liang Y, Zhang H, Song X, Yang Q (2020). Metastatic heterogeneity of breast cancer: molecular mechanism and potential therapeutic targets. Semin Cancer Biol.

[8] Tabariès S, McNulty A, Ouellet V, Annis MG, Dessureault M, Vinette M (2019). Afadin cooperates with Claudin-2 to promote breast cancer metastasis. Genes Dev.

[9] Kapranov P, Cheng J, Dike S, Nix DA, Duttagupta R, Willingham AT (2007). RNA maps reveal new RNA classes and a possible function for pervasive transcription. Science..

[10] Chen L-L, Yang L (2015). Regulation of circRNA biogenesis. RNA Biol.

[11] Jeck WR, Sorrentino JA, Wang K, Slevin MK, Burd CE, Liu J (2013). Circular RNAs are abundant, conserved, and associated with ALU repeats. RNA..

[12] Kristensen LS, Andersen MS, Stagsted LVW, Ebbesen KK, Hansen TB, Kjems J (2019). The biogenesis, biology and characterization of circular RNAs. Nat Rev Genet.

[13] Zhou W-Y, Cai Z-R, Liu J, Wang D-S, Ju H-Q, Xu R-H (2020). Circular RNA: metabolism, functions and interactions with proteins. Mol Cancer.

[14] Lei M, Zheng G, Ning Q, Zheng J, Dong D (2020). Translation and functional roles of circular RNAs in human cancer. Mol Cancer.

[15] Vo JN, Cieslik M, Zhang Y, Shukla S, Xiao L, Zhang Y (2019). The landscape of circular RNA in Cancer. Cell..

[16] Yu T, Wang Y, Fan Y, Fang N, Wang T, Xu T (2019). CircRNAs in cancer metabolism: a review. J Hematol Oncol.

[17] Meng S, Zhou H, Feng Z, Xu Z, Tang Y, Li P (2017). CircRNA: functions and properties of a novel potential biomarker for cancer. Mol Cancer.

[18] Wang Z, Yang L, Wu P, Li X, Tang Y, Ou X (2022). The circROBO1/KLF5/FUS feedback loop regulates the liver metastasis of breast cancer by inhibiting the selective autophagy of afadin. Mol Cancer.

[19] Dudekula DB, Panda AC, Grammatikakis I, De S, Abdelmohsen K, Gorospe M (2016). CircInteractome: a web tool for exploring circular RNAs and their interacting proteins and microRNAs. RNA Biol.

[20] Enright AJ, John B, Gaul U, Tuschl T, Sander C, Marks DS (2003). MicroRNA targets in Drosophila. Genome Biol.

[21] Vejnar CE, Zdobnov EM (2012). MiRmap: comprehensive prediction of microRNA target repression strength. Nucleic Acids Res.

[22] Maragkakis M, Reczko M, Simossis VA, Alexiou P, Papadopoulos GL, Dalamagas T (2009). DIANA-microT web server: elucidating microRNA functions through target prediction. Nucleic Acids Res.

[23] Chen Y, Wang X (2020). miRDB: an online database for prediction of functional microRNA targets. Nucleic Acids Res.

[24] Agarwal V, Bell GW, Nam J-W, Bartel DP (2015). Predicting effective microRNA target sites in mammalian mRNAs. eLife..

[25] Jia L, Zhou Z, Liang H, Wu J, Shi P, Li F (2016). KLF5 promotes breast cancer proliferation, migration and invasion in part by upregulating the transcription of TNFAIP2. Oncogene..

[26] Qin J, Zhou Z, Chen W, Wang C, Zhang H, Ge G (2015). BAP1 promotes breast cancer cell proliferation and metastasis by deubiquitinating KLF5. Nat Commun.

[27] Győrffy B (2021). Survival analysis across the entire transcriptome identifies biomarkers with the highest prognostic power in breast cancer. Comput Struct Biotechnol J.

[28] Jézéquel P, Gouraud W, Ben Azzouz F, Guérin-Charbonnel C, Juin PP, Lasla H (2021). Bc-GenExMiner 4.5: new mining module computes breast cancer differential gene expression analyses. Database (Oxford).

[29] Mei S, Qin Q, Wu Q, Sun H, Zheng R, Zang C (2017). Cistrome data browser: a data portal for ChIP-Seq and chromatin accessibility data in human and mouse. Nucleic Acids Res.

[30] Castro-Mondragon JA, Riudavets-Puig R, Rauluseviciute I, Lemma RB, Turchi L, Blanc-Mathieu R (2022). JASPAR 2022: the 9th release of the open-access database of transcription factor binding profiles. Nucleic Acids Res.

[31] Li J-H, Liu S, Zhou H, Qu L-H, Yang J-H (2014). starBase v2.0: decoding miRNA-ceRNA, miRNA-ncRNA and protein-RNA interaction networks from large-scale CLIP-Seq data. Nucleic Acids Res.

[32] Bray F, Ferlay J, Soerjomataram I, Siegel RL, Torre LA, Jemal A (2018). Global cancer statistics 2018: GLOBOCAN estimates of incidence and mortality worldwide for 36 cancers in 185 countries. CA Cancer J Clin.

[33] Ng WL, Mohd Mohidin TB, Shukla K. Functional role of circular RNAs in cancer development and progression. RNA Biol. 2018;15(8):995–1005. 10.1080/15476286.2018.1486659 PMC6259826 29954251

[34] Meng J, Chen S, Han J-X, Qian B, Wang X-R, Zhong W-L (2018). Twist1 regulates Vimentin through Cul2 circular RNA to promote EMT in hepatocellular carcinoma. Cancer Res.

[35] Conn SJ, Pillman KA, Toubia J, Conn VM, Salmanidis M, Phillips CA (2015). The RNA binding protein quaking regulates formation of circRNAs. Cell..

[36] Wang R, Zhang S, Chen X, Li N, Li J, Jia R (2018). EIF4A3-induced circular RNA MMP9 (circMMP9) acts as a sponge of miR-124 and promotes glioblastoma multiforme cell tumorigenesis. Mol Cancer.

[37] Zheng X, Huang M, Xing L, Yang R, Wang X, Jiang R (2020). The circRNA circSEPT9 mediated by E2F1 and EIF4A3 facilitates the carcinogenesis and development of triple-negative breast cancer. Mol Cancer.

[38] Han J, Meng J, Chen S, Wang X, Yin S, Zhang Q (2019). YY1 complex promotes quaking expression via super-enhancer binding during EMT of hepatocellular carcinoma. Cancer Res.

[39] Errichelli L, Dini Modigliani S, Laneve P, Colantoni A, Legnini I, Capauto D (2017). FUS affects circular RNA expression in murine embryonic stem cell-derived motor neurons. Nat Commun.

[40] Lagier-Tourenne C, Polymenidou M, Cleveland DW (2010). TDP-43 and FUS/TLS: emerging roles in RNA processing and neurodegeneration. Hum Mol Genet.

[41] Han K, Wang F-W, Cao C-H, Ling H, Chen J-W, Chen R-X (2020). CircLONP2 enhances colorectal carcinoma invasion and metastasis through modulating the maturation and exosomal dissemination of microRNA-17. Mol Cancer.

[42] Su J, Wang Q, Liu Y, Zhong M (2014). miR-217 inhibits invasion of hepatocellular carcinoma cells through direct suppression of E2F3. Mol Cell Biochem.

[43] Zhou C, Chen Y, He X, Zheng Z, Xue D (2020). Functional implication of Exosomal miR-217 and miR-23b-3p in the progression of prostate Cancer. Onco Targets Ther.

[44] Li W, Yang X, Shi C, Zhou Z (2020). Hsa_circ_002178 promotes the growth and migration of breast Cancer cells and maintains Cancer stem-like cell properties through regulating miR-1258/KDM7A Axis. Cell Transplant.

[45] Liu C, Zhang Z, Qi D (2019). Circular RNA hsa_circ_0023404 promotes proliferation, migration and invasion in non-small cell lung cancer by regulating miR-217/ZEB1 axis. OTT..

[46] Ben-Porath I, Thomson MW, Carey VJ, Ge R, Bell GW, Regev A (2008). An embryonic stem cell–like gene expression signature in poorly differentiated aggressive human tumors. Nat Genet.

[47] Takagi K, Miki Y, Onodera Y, Nakamura Y, Ishida T, Watanabe M (2012). Krüppel-like factor 5 in human breast carcinoma: a potent prognostic factor induced by androgens. Endocr Relat Cancer.

[48] Tong D, Czerwenka K, Heinze G, Ryffel M, Schuster E, Witt A (2006). Expression of *KLF5* is a prognostic factor for disease-free survival and overall survival in patients with breast Cancer. Clin Cancer Res.

[49] Liu R, Shi P, Zhou Z, Zhang H, Li W, Zhang H (2018). Krüpple-like factor 5 is essential for mammary gland development and tumorigenesis: KLF5 and mammary gland development. J Pathol.

[50] Chen C, Benjamin MS, Sun X, Otto KB, Guo P, Dong X-Y (2006). KLF5 promotes cell proliferation and tumorigenesis through gene regulationin the TSU-Pr1 human bladder cancer cell line. Int J Cancer.

[51] Long X, Singla DK (2013). Inactivation of Klf5 by zinc finger nuclease downregulates expression of pluripotent genes and attenuates colony formation in embryonic stem cells. Mol Cell Biochem.

[52] Zheng H-Q, Zhou Z, Huang J, Chaudhury L, Dong J-T, Chen C (2009). Krüppel-like factor 5 promotes breast cell proliferation partially through upregulating the transcription of fibroblast growth factor binding protein 1. Oncogene..

[53] Jia X, Chen H, Ren Y, Dejizhuoga, Gesangyuzhen, Gao N (2021). BAP1 antagonizes WWP1-mediated transcription factor KLF5 ubiquitination and inhibits autophagy to promote melanoma progression. Exp Cell Res.

[54] Tang J, Li Y, Sang Y, Yu B, Lv D, Zhang W (2018). LncRNA PVT1 regulates triple-negative breast cancer through KLF5/beta-catenin signaling. Oncogene..

[55] Gupta GP, Massagué J (2006). Cancer metastasis: building a framework. Cell..

[56] Heerboth S, Housman G, Leary M, Longacre M, Byler S, Lapinska K (2015). EMT and tumor metastasis. Clin Transl Med.

[57] Brabletz T, Kalluri R, Nieto MA, Weinberg RA (2018). EMT in cancer. Nat Rev Cancer.

[58] Hugo H, Ackland ML, Blick T, Lawrence MG, Clements JA, Williams ED (2007). Epithelial—mesenchymal and mesenchymal—epithelial transitions in carcinoma progression. J Cell Physiol.

[59] Zhang B, Li Y, Wu Q, Xie L, Barwick B, Fu C (2021). Acetylation of KLF5 maintains EMT and tumorigenicity to cause chemoresistant bone metastasis in prostate cancer. Nat Commun.

[60] Guo H, Ge Y, Li X, Yang Y, Meng J, Liu J (2017). Targeting the CXCR4/CXCL12 axis with the peptide antagonist E5 to inhibit breast tumor progression. Sig Transduct Target Ther.

[61] Liu D, Guo P, McCarthy C, Wang B, Tao Y, Auguste D (2018). Peptide density targets and impedes triple negative breast cancer metastasis. Nat Commun.

[62] Yang F, Takagaki Y, Yoshitomi Y, Ikeda T, Li J, Kitada M (2019). Inhibition of Dipeptidyl Peptidase-4 Accelerates Epithelial–Mesenchymal Transition and Breast Cancer Metastasis via the CXCL12/CXCR4/mTOR Axis. Cancer Res.

[63] Correia AL, Guimaraes JC, Auf der Maur P, De Silva D, Trefny MP, Okamoto R (2021). Hepatic stellate cells suppress NK cell-sustained breast cancer dormancy. Nature..

