# Supplementary material for: The FUS/circEZH2/KLF5/ feedback loop contributes to CXCR4-induced liver metastasis of breast cancer by enhancing epithelial-mesenchymal transition
Source: Mol Cancer. 2022 Oct 12;21:198. doi: 10.1186/s12943-022-01653-2 (PMC9555172; doi:10.1186/s12943-022-01653-2)
Supplement: Supplementary file 1 — Additional file 1. Supplemental Figure 1 [file 12943_2022_1653_MOESM1_ESM.pdf]

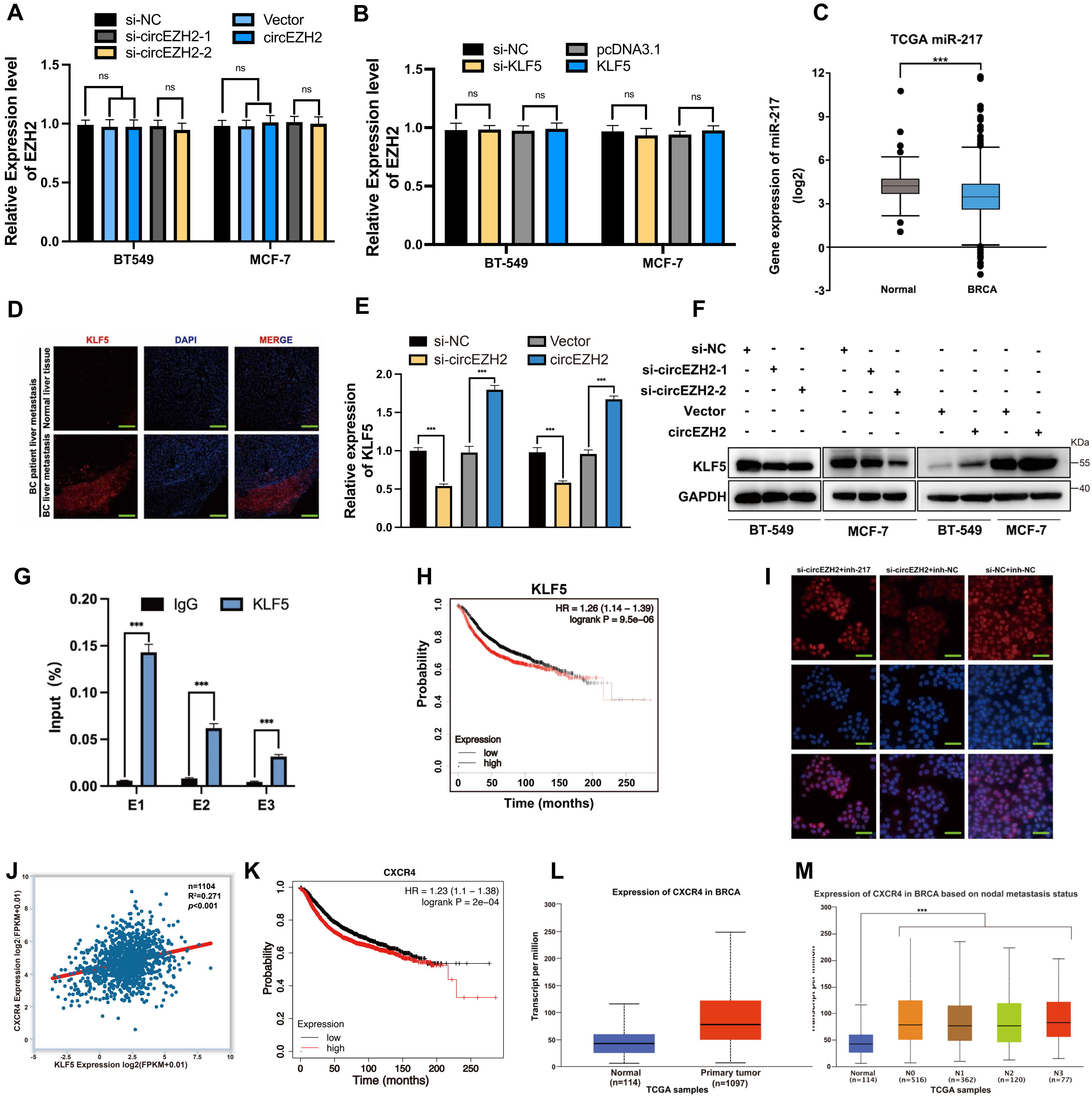

A
si-NC
si-circEZH2-1
si-circEZH2-2
Vector
circEZH2
Relative Expression level of EZH2
BT549
MCF-7
B
si-NC
si-KLF5
pcDNA3.1
KLF5
Relative Expression level of EZH2
BT-549
MCF-7
C
TCGA miR-217
Gene expression of miR-217 (log2)
Normal
BRCA
D
KLF5
DAPI
MERGE
BC patient liver metastasis
Normal liver tissue
BC liver metastasis
E
si-NC
si-circEZH2
Vector
circEZH2
Relative expression of KLF5
F
si-NC
si-circEZH2-1
si-circEZH2-2
Vector
circEZH2
KDa
KLF5
GAPDH
BT-549
MCF-7
G
IgG
KLF5
Input (%)
E1
E2
E3
H
KLF5
HR = 1.26 (1.14 – 1.39)
logrank P = 9.5e−06
Probability
Expression
low
high
Time (months)
I
si-circEZH2+inh-217
si-circEZH2+inh-NC
si-NC+inh-NC
J
n=1104
R²=0.271
p<0.001
CXCR4 Expression log2(FPKM+0.01)
KLF5 Expression log2(FPKM+0.01)
K
CXCR4
HR = 1.23 (1.1 – 1.38)
logrank P = 2e−04
Probability
Expression
low
high
Time (months)
L
Expression of CXCR4 in BRCA
Transcript per million
Normal (n=114)
Primary tumor (n=1097)
TCGA samples
M
Expression of CXCR4 in BRCA based on nodal metastasis status
Normal (n=114)
N0 (n=516)
N1 (n=362)
N2 (n=120)
N3 (n=77)
TCGA samples

a RT-qPCR was performed to determine the expression change of EZH2 after overexpression or knockdown of circEZH2.
b After overexpression or knockdown of KLF5, the expression of EZH2 identified by RT-qPCR.
c TCGA database analysis showed that miR-217 was downregulated in BRCA samples compared with normal tissues.
d Immunofluorescence of liver metastasis slide from human BC patient was conducted to explore the expression of KLF5 on metastases compared with its adjacent normal liver tissue (Scale Bar 200μm).
e-f RT-qPCR and Western blot were performed to determine the expression of KLF5 after overexpression or knockdown of circEZH2 in RNA or protein level.
g ChIP assays were performed to find KLF5 enrichment efficiency of E1, E2, and E3 regions..
h High expression of KLF5 indicated with poor prognosis in BRCA patients according to TCGA database.
i IF was performed to showed that miR-217-5p inhibitor could reserves downregulation of KLF5 induced via circEZH2 (Scale bar 20μm).
j STARBASE correlation analysis showed that CXCR4 was positive correlated with KLF5 transcript.
k High expression of CXCR4 was related with poor prognosis in BRCA patients according to TCGA database.
l-m TCGA database showed that CXCR4 was upregulated in Primary tumor compared with normal tissues while CXCR4 was upregulated in different stages of BC compared to normal stage.
The data are showed as the mean ± SD and all experiments were repeated at least three times, ns no significant, *P < 0.05 **P < 0.01, ***P < 0.001
