# Supplementary material for: The FUS/circEZH2/KLF5/ feedback loop contributes to CXCR4-induced liver metastasis of breast cancer by enhancing epithelial-mesenchymal transition
Source: Mol Cancer. 2022 Oct 12;21:198. doi: 10.1186/s12943-022-01653-2 (PMC9555172; doi:10.1186/s12943-022-01653-2)
Supplement: Supplementary file 2 — Additional file 2: Table S1. Sequences of siRNAs used in this study [file 12943_2022_1653_MOESM2_ESM.docx]

**Table S1 Sequences of siRNAs used in this study**

| **Definition** | **sequences** |
| --- | --- |
| si-circEZH2-1 | 5'-CAGCAGAAUUUUAUGAAUA-3' |
| si-circEZH2-2 | 5'-CAGAAUUUUAUGAAUAAUC-3' |
| si-NC | 5'-UUCUCCGAACGUGUCACGU-3' |
| si-FUS | 5'-CAGAGUUACAGUGGUUAUA-3' |
| si-KLF5 | 5’-GCAGACUGCAGUGAAACAA-3’ |
| si-PTBP1 | 5’-CAGUUUACCUGUUUUUAAA-3’ |
| si-EIF4A3 | 5’-CCAACUACCUGAACGAUGA-3’ |
| si-U2AF65 | 5’-CGAGCAAUCAAGCAGAUCA-3’ |
