# Supplementary material for: The FUS/circEZH2/KLF5/ feedback loop contributes to CXCR4-induced liver metastasis of breast cancer by enhancing epithelial-mesenchymal transition
Source: Mol Cancer. 2022 Oct 12;21:198. doi: 10.1186/s12943-022-01653-2 (PMC9555172; doi:10.1186/s12943-022-01653-2)
Supplement: Supplementary file 3 — Additional file 3: Table S2. Primer sequences used in RT-qPCR and PCR analysis [file 12943_2022_1653_MOESM3_ESM.docx]

**Table S2 Primer sequences used in RT-qPCR and PCR analysis**

| **Gene** | **Primer sequences** |
| --- | --- |
| GAPDH | F: 5'-GAAGGTGAAGGTCGGAGTC-3' |
|  | R: 5'-GAAGATGGTGATGGGATTTC-3' |
| U6 | F: 5'-CGAGCACAGAATCGCTTCA-3' |
|  | R: 5'-CTCGCTTCGGCAGCACATAT-3' |
| circEZH2 | F: 5'- TCTTGGTCTCCCCTACAGCA-3' |
|  | R: 5'- GAGCTGTCTCAGTCGCATGT-3' |
| EZH2 | F: 5'- GTACACGGGGATAGAGAATGTGG-3' |
|  | R: 5'- GGTGGGCGGCTTTCTTTATCA-3' |
| FUS | F: 5'-CAAGGCCTGGGTGAGAATGT-3' |
|  | R: 5'-TTGCCTCTCCCTTCAGCTTG-3' |
| KLF5 | F: 5'-CCTGGTCCAGACAAGATGTGA-3' |
|  | R: 5'-GAACTGGTCTACGACTGAGGC-3' |
| FUS E1 | F: 5'-CTGTGACTCCAGTTTCGTCC-3' |
|  | R: 5'-CTCTCTCCTCGGCAGGACTA-3' |
| FUS E2 | F:5’-GAGGTCAACCCTCTCTGGTC-3’ |
|  | R:5’-GACCTGAAAGACAGCAGGGATT-3’ |
| FUS E3 | F:5’-AAGTCAACCTCAGGGCGCAG-3’ |
|  | R:5’-CTTTCACCCAGGATGGCGAG-3’ |
| hsa_circ_0056037 | F:5’-AGCCATCAAGCCCAAGACTG-3’ |
|  | R:5’-TGTTCACTGGTGTCTGCTGAT-3’ |
| hsa_circ_0060949 | F:5’-ATGCACCACTTGGAACAGTTT-3’ |
|  | R:5’-TGCTGAGTCACGAGAACACG-3’ |
| hsa_circ_0063300 | F: 5'-CCACCAAGCGCCAGAAGTAT-3' |
|  | R: 5'-CATGAGGAAGTTGTCGGGCT-3' |
| CXCR4 E1 | F:5’-ACCCGCAAACAGCAGGGTC-3’ |
|  | R:5’-GCCGCAGCCAACAAACTGAA-3’ |
| CXCR4 E2 | F:5’- TTAAACGTCTGACCCCCACCC-3’ |
|  | R: 5'- CTTCGGTCCCCATACTCGGC-3' |
| CXCR4 E3 | F: 5'- CCTCGGTCCCAGCTATCTCC-3' |
|  | R:5’- GGGTGGGGGTCAGACGTTTA-3’ |
| HA-circEZH2 | F:5’-AATCTGGAACATCATATGGATA-3’ |
|  | R:5’-GAGCTGTCTCAGTCGCATGT-3’ |
| HA-preEZH2 | F:5’-TATCCATATGATGTTCCAGATT-3’ |
|  | R:5’-ACATGCGACTGAGACAGCTC-3’ |
