# Supplementary material for: The FUS/circEZH2/KLF5/ feedback loop contributes to CXCR4-induced liver metastasis of breast cancer by enhancing epithelial-mesenchymal transition
Source: Mol Cancer. 2022 Oct 12;21:198. doi: 10.1186/s12943-022-01653-2 (PMC9555172; doi:10.1186/s12943-022-01653-2)
Supplement: Supplementary file 4 — Additional file 4. Supplemental Method [file 12943_2022_1653_MOESM4_ESM.docx]

**Supplemental Method**

**Microarray analysis**

Invitrogen's TRIzol reagent (CA, USA) was used to extract total RNA from three pairs of fresh frozen BC primary tissues and three pairs of matched liver metastatic tissues. Microarray hybridization was performed following Arraystar's standard protocols (Rockville, MD, USA).

**Fluorescence in situ hybridization (FISH)**

CircEZH2 was identified in BC cells and tissues by Cy3 labeling (5'-CTACAGCAGAATTTTATGAATAATCATGGGCCAGACTGGG-3') probe (GenePharm, Shanghai, China). Following the protocol of the manufacturer, FISH assays were carried out using the Fluorescent In Situ Hybridization Kit (GenePharm, Shanghai, China). A fluorescence microscope (Olympus, Japan) was used to capture images of FISH assay results.

**EdU assay**

Before transfection, cells were seeded on coverslips in 24-well plates and incubated according to standard culture conditions. 72 hours later, adding EdU to the culture medium and incubating in 37℃ for 2 hours. Then, fixed by 4% Paraformaldehyde for 15 min in room temperature. After washing off paraformaldehyde, adding 0.3% Triton X-100 (Invitrogen, USA) into the plates and incubated for 20min. Next, adding Click Additive Solution according to the instruction provided by the commercial protocol (BeyoClick™ EdU-555, Shanghai, China), then incubating at room temperature for 30 minutes, protected in dark. Finally, stain the nucleus with Hoechst 33342, and took images under a fluorescent microscope (Olympus, Japan).

**Sanger sequencing**

Specific divergent primers targeting circEZH2 was designed to PCR the sequence of special junction site of circEZH2 by Takara PrimeSTAR^®^ Max DNA Polymerase (Takara, Japan), and performed Sanger sequencing (Ruibiotech, Guangzhou, China) of the amplification results to identify the special sequence of circEZH2 junction site.

**Western blot analysis**

An extract of the proteins was separated on 6-12% SDS-PAGE gel, followed by the transfer of the isolated proteins to PVDF membranes (Milliore, MA, USA). Incubation with primary antibodies was performed overnight at 4°C using 5% skim milk. Then secondary antibodies were incubated with the membranes for 1 hour. Finally, the protein bands were detechnified with chemiluminescence. The primary antibodies used in the study were: anti-KLF5 (21017-1-AP, Proteintech, China), anti-FUS (11570-1-AP, Proteintech, China), anti-mTOR (66888-1-Ig, Proteintech, China), anti-p-mTOR(67778-1-Ig, Proteintech, China), anti-AKT (#4691, CST, USA), anti-p-AKT (#4060, CST, USA), anti-PI3K(20584-1-AP, Proteintech, China), anti-GAPDH (60004-1-Ig, Proteintech, China), anti-E-Cadherin (#3195, CST, USA), anti-N-Cadherin (#13116, CST, USA), anti-Vimentin (#5741, CST, USA), anti-CXCR4 antibody (60042-1-Ig, Proteintech, China), anti-HA Mouse antibody (66006-2-Ig, Proteintech, China), anti-Flag Mouse antibody(66008-3-Ig, Proteintech, China). The secondary antibodies that we used in this study: anti-Rabbit-IgG HRP-link antibody (#7074, CST, USA), anti-Mouse-IgG HRP-link antibody (#7076, CST, USA).

**Plasmid construction**

A cricEZH2 overexpression vector was constructed by cloning the full length of human circEZH2 into a PLCDH-ciR vector (GENESEED BIOTECH, Guangzhou, China). To establish stably transfected overexpressed circEZH2 BC cells, circEZH2 or a relative control vector were transfected into HEK293T cells with packaging assistant vectors to generate a lentivirus that overexpresses circEZH2. Afterward, lentivirus was used to infect and overexpress circEZH2, with puromycin as a screening method to show circEZH2 stably expressed cell lines. Our overexpression vectors for KLF5 and FUS were constructed by cloning the full-length, human-derived sequences of FUS and KLF 5 into pcDNA3.1(+) (Thermofisher, MA, USA). Additionally, we cloned the promoter of human FUS into the pEZX-FR01 vector (GeneCopeia, Guangzhou, China). Sanger sequencing was used to confirm the correctness of all sequences in the vectors above. pc-HA-EZH2 was constructed by cloning a 2000bp fragment upstream of EZH2 exon 2 and a 2000bp fragment downstream of EZH2 exon 4 with an HA tag inserted into the 5' end of exon 2. Following the integration of the above recombination sequence into pcDNA3.1(+), we created pc-HA-EZH2.

**Immunohistochemistry (IHC) and immunofluorescence (IF)**

IHC and IF assays were performed as previously reported. For IHC assays, paraffin tissue slides were incubated with the anti-KLF5 primary antibody (21017-1-AP, Proteintech, China) (1:100 dilution). Images were captured by Nikon Eclipse 80i microscope (Nikon, Tokyo, Japan). As for IF experiment, cells or tissue slides were incubated with anti-KLF5 body (21017-1-AP, Proteintech, China), anti-E-Cadherin (#3195, CST, USA), anti-N-Cadherin (#13116, CST, USA), anti-Vimentin (#5741, CST, USA), anti-CXCR4 antibody (60042-1-Ig, Proteintech, China) at 4 °C overnight, then incubated with fluorescein CoraLite594–conjugated secondary antibodies (SA00013-4, Proteintech, China) and imaged by using Olympus fluorescence microscope (Olympus, Japan).

**RNase R, and nuclear-cytoplasmic fractionation, actinomycin D assays**

A 30-minute RNase R assay was performed with 4 units per gram of RNase R (Geenseed Biotech, Guangzhou, China). RNA from nuclear and cytoplasmic fractions of cell lines was separated according to protocols using Invitrogen's PARIS Kit. Moreover, after treatment with 120ng/ml actinomycin D (Solarbio, Shanghai, China) for 4hours, 8hours, 12 hours, and 24 hours, the RNA of BC cell lines was extracted.

**Dual-luciferase reporter assay**

The sequences of circEZH2 or KLF5 3’UTR that contained the wild-type (WT) or mutant-type (MT) binding sites of hsa-miR-217-5p and the promotor region of FUS that contained the wild-type (WT) or mutant-type (MT) binding sites of KLF5 were all produced by Ruibiotech (Beijing, China). Then we recombined the MT or WT sequences of circEZH2 or KLF5 3’UTR into pmirGLO (Promega, USA). Next, co-transfections were performed with the corresponding vectors and mimics-NC/mimics or inhibitor-NC/inhibitors of hsa-miR-217-5p in HEK293T cells, respectively. Meanwhile, the MT or WT sequences of FUS promotor were recombined into pEZX-FR01 (GeneCopoeia, MD, USA). Next, co-transfection with vectors constructed above with KLF-overexpression vectors or KLF5-siRNA in HEK293T cells as well. The relative activities of Firefly and Renilla luciferase of all the transfections above were analyzed by Dual-Luciferase Reporter Assay Kit (Promega, WI,USA) after 48h-incubation while Renilla was an internal reference.

**RNA pull-down assay**

The biotinylated circEZH2 probe (5’—3’) and control probe (5’—3’) were designed and synthesized by Ribobio (Guangzhou, China) while the probes of pre-EZH2 were transcribed in vitro via MAXIscriptTM T7 Transcription Kit (Invitrogen, CA, USA). After that, RNA-protein Pull Down Kit (Thermofisher, MA, USA) was used to conduct the following procedures according to the standard protocols. Briefly, after incubation of biotinylated probe with streptavidin magnetic beads for half an hour, magnetic beads-probe complex was incubated with cellular lysates at 4°C overnight and the resultants were eluted for RNA analysis by TRIzol (Invitrogen, CA, USA) or protein analysis by SDS-PAGE loading buffer (Beyotime, Shanghai, China). The enrichment of hsa-miR-217-5p was detected by RT-qPCR while the abundance of protein was analyzed by western blotting.

**Cell proliferation, wound healing assays, transwell assays**

As previously reported, colony formation assays, EdU assays, CCK-8 assays, and transwell assays were performed [19, 20].

**siRNAs and cell transfection**

To knock down circEZH2, siRNAs that aimed at the back-splicing junction of circEZH2 (si-circEZH2-1, si-circEZH2-2) and siRNA-NC were designed and synthesized GenePharma (Shanghai, China). The siRNAs of FUS, KLF5, the mimics and inhibitors of hsa-miR-217-5p were designed and synthesized from GenePharma (Shanghai, China) while NC of mimics and inhibitors were used as controls. Transfections were performed by Lipofectamine 3000 (Invitrogen, CA, USA) according to the standard protocols. The siRNA sequences used in this article were listed in Supplementary Table 1.

**Biotinylation probe synthesis and RNA pull-down assay**

The biotinylated circEZH2 probe (5′-TTTTATGAATAATCATGGGC-3′) and control probe (5′-GAAACTGCTCGGAACGTTAA-3′) were designed by RiboBio (Guangzhou, China). In vitro transcription of pre-EZH2 probes was accomplished with the MAXIscript T7 transcription kit (Invitrogen, CA, USA). Afterwards, we performed RNA pulldown using the RNA-Protein Pull Down Kit (Thermo Fisher, MA, USA). RT-qPCR was used to determine miR-217-5p enrichment while western blotting was used to determine abundant protein levels.

**Chromatin immunoprecipitation (ChIP)**

We used the magnetic beads in conjunction with the Magnetic Bead ChIP Kit (Thermo Fisher, MA, USA), following the standard protocol. The final enriched DNA samples were then subjected to qPCR analysis.

**RNA immunoprecipitation (RIP)**

The Magna RIP Kit (Millipore, MA, USA) was taken to conduct RIP assays following the manufacture’s standard protocols. The co-precipitated RNA resultants were extracted and purified via TRIzol (Invitrogen, CA, USA) and analyzed by RT-qPCR.
